# Association of Intersectional Anticipated Discrimination with Mental Health Among Immigrant Latinos

**DOI:** 10.1089/heq.2024.0072

**Published:** 2024-11-26

**Authors:** Cassandra Parent, Gabriel Ramírez, Cui Yang, Suzanne M. Grieb, Ronald E. Saxton, Diego A. Martínez, Kathleen R. Page

**Affiliations:** ^1^ Department of Infectious Diseases, Johns Hopkins University School of Medicine, Baltimore, Maryland, USA.; ^2^ Department of Health Behavior, Society, and Policy, Rutgers School of Public Health, Piscataway, New Jersey, USA.; ^3^ Department of Pediatrics, Johns Hopkins University School of Medicine, Baltimore, Maryland, USA.; ^4^ School of Industrial Engineering, Pontificia Universidad Católica de Valparaíso, Valparaíso, Chile.; ^5^ Department of Emergency Medicine, Johns Hopkins University School of Medicine, Baltimore, Maryland, USA.

**Keywords:** intersectionality, discrimination, mental health, Latinos

## Abstract

**Introduction:**

Anticipating discrimination can lead to increased vigilance, which acts as a potential stressor similar to actual discrimination experiences. However, there is limited understanding of how discrimination and anticipated discrimination affect Latinos with intersecting identities, particularly those who are immigrants. Using a cross-sectional survey, we examine the association between intersectional anticipated discrimination and mental health among immigrant Latinos.

**Methods:**

We conducted a cross-sectional survey through the Rapid Acceleration of Diagnostics-Underserved Populations initiative (March 2022–May 2023). Participants were foreign-born adults who self-identified as Latino or Hispanic. The exposure measure used the Intersectional Anticipated Discrimination Scale, and outcomes measures included 2-item screens for anxiety (Generalized Anxiety Disorder screener [GAD-2]) and depression (Patient Health Questionnaire [PHQ-2]) and a 3-item screen for hazardous alcohol consumption (Alcohol Use Disorders Identification Test).

**Results:**

A total of 810 participants completed the survey, of whom 66.7% were uninsured. Among them, 25.2% screened positive for anxiety, 18.1% for depression, and 20.2% for hazardous alcohol consumption. Positive screening for anxiety and depression was associated with higher levels of anticipated discrimination (GAD-2 adjusted odds ratio [AOR] = 1.05, 95% confidence interval [CI]: 1.03, 1.07; PHQ-2 AOR = 1.05, 95% CI: 1.03, 1.07). A dose–response relationship was observed with higher levels of anticipated discrimination and higher PHQ-2 and GAD-2 scores.

**Conclusions:**

Anticipated intersectional discrimination was associated with symptoms of anxiety and depression in immigrant Latinos. Prioritizing culturally competent care that recognizes the heterogeneity of the Latino population, enhancing community support, and implementing targeted policy interventions are imperative steps toward promoting mental health equity among this population.

## Introduction 

In the United States, Latinos report high levels of discrimination and violence.^1^ In addition to facing prejudice based on race and ethnicity, many Latinos encounter bias due to low socioeconomic status, unfair stereotypes associated with reliance on social welfare, legal status, language, and anti-immigrant attitudes.^2^ Immigration status, especially for those without documentation, can heighten this risk. Undocumented immigrants may encounter difficulties accessing basic services and legal protection, and discrimination against them may be compounded by negative portrayals in the media, public discourse, and political rhetoric that can shape attitudes toward them.^2,3^ According to a survey conducted by the Pew Research Center in 2018, 38% of Latinos in the United States reported experiencing discrimination, with more incidents reported by Spanish-dominant, immigrant, and second-generation Latinos.^4^

The combined effects of minority status, previous or current trauma, legal status, limited access to care, chronic financial stress, and participation in the informal economy where unsafe working conditions, wage theft, and workplace discrimination are common can increase the risk of stress and unhealthy coping mechanisms.^5,6^ Structural forms of discrimination against immigrants are associated with detrimental mental health outcomes at a population level. For example, Latino adults and children living in states with more exclusionary immigration policies experience worse mental health outcomes than those in states with more inclusive policies.^7,8^ On the contrary, policies can mitigate stress and promote well-being. Children born to Latino mothers eligible for the Deferred Action for Childhood Arrivals program, which protects recipients from deportation and grants temporary work permits, exhibit reduced rates of anxiety and depression compared with children of ineligible mothers, highlighting the intergenerational impact of structural discrimination on mental health.^9^

The intersectionality framework offers an approach to study the role of societal structures in perpetuating inequalities among individuals with intersecting marginalized identities. Decades ago, Black feminist scholars created this framework suggesting that various factors such as race, ethnicity, gender identity, and socioeconomic status lead to intersectional discrimination at different levels—individual, interpersonal, and structural.^10,11^ The experience of navigating intersecting identities can expose marginalized populations to heightened risk of social marginalization, isolation, and chronic stress- all of which can lead to adverse mental health outcomes.^6,12^ Intersectional discrimination in various environments, including health care, can contribute to trauma and further exacerbate disparities.^13^ Furthermore, internalized intersectional stigma may perpetuate negative stereotypes, and impact self-esteem and overall well-being.^14^

While intersectional discrimination highlights the compounded impact of multiple, overlapping identities on individuals’ experiences, it also informs the way people anticipate discrimination in different domains, such as health care, or housing. This anticipation is shaped by the awareness that they may face bias not just for one aspect of their identity, but for the combined effect of several marginalized identities. Anticipating discrimination or prejudice can lead to increased vigilance, which acts as a potential stressor similar to actual discrimination experiences.^15^ Qualitative studies highlight the impact of anticipatory discrimination and concerns about deportation on chronic stress, family fragmentation, and social relationships among immigrant Latina women.^16^ This type of anticipatory discrimination also impacts Latinos who are U.S. citizens. For example, U.S.-born adolescent Latinos who worry about the personal repercussions of immigration policy have higher levels of anxiety.^17^

Although studies have explored the negative effects of discrimination on health, primarily based on individual attributes, notably race or ethnicity, there are limited studies on the negative impact of anticipated intersectional discrimination and its correlation with mental health and well-being among Latino immigrants. The purpose of this study was to assess the association between anticipated intersectional discrimination and anxiety, depression, or hazardous alcohol use among a sample of Latino immigrant adults using the validated Intersectional Anticipated Discrimination Scale (InDI-A). The InDI-A assesses discrimination across various contexts by capturing participants’ concerns about experiencing discrimination “because of who I am.” This phrasing allows for the acknowledgment of multiple intersecting identities and how they collectively contribute to anticipated discrimination, rather than limiting the understanding to a single aspect of identity (e.g., discrimination solely due to race). By focusing on the broad experience of discrimination, the InDI-A reflects the complex, multifaceted nature of discrimination that individuals with intersecting marginalized identities may anticipate across different domains, such as housing and health care.

## Methods

### Study design

The data used for this analysis was collected using a cross-sectional survey study design as part of the Rapid Acceleration of Diagnostics–Underserved Populations (RADx-UP) initiative. Participants were recruited via convenience sampling at community venues, through social marketing, and network-based referrals. Participant inclusion criteria included: self-identified as Latino or Hispanic, 18 years old or older, not born in the United States, and living in Maryland. Data collection took place from March 16, 2022, to May 3, 2023. Surveys, offered in English or Spanish, were administered online, over the phone, or in person by bilingual research staff. All project activities received approval from the institutional review board at Johns Hopkins University.

### Measures

Validated screening tools were used to screen mental health outcomes, including the Generalized Anxiety Disorder screener (GAD-2) for anxiety, the Patient Health Questionnaire (PHQ-2) for depression, and the 3-item Alcohol Use Disorders Identification Test (AUDIT-C) for active alcohol abuse or hazardous alcohol consumption. Positive screens were defined as a cutoff score ≥ 3 for GAD-2 and PHQ-2 and for AUDIT-C ≥ 3 for women and ≥ 4 for men.^18–20^

Anticipated discrimination was assessed using the InDI-A, one of the three measures of the Intersectional Discrimination Index (InDI), designed to support social research analysis with a focus on the intersection of social identities and positions.^21^ The InDI-A assesses discrimination across a range of intersectional marginalized social identities by asking participants to endorse discriminatory concerns “because of who I am” rather than attributing an experience of discrimination to a single axis of one’s identity (e.g., discrimination due to race). Participants respond to nine items reflecting discrimination in different settings including health care, employment, housing, and banking; by specific people including discrimination by a supervisor and discrimination by police; and concerns about violence, harassment, and relationships. Individual items are scored on a 5-point Likert scale ranging from strongly disagree (0) to strongly agree,^4^ with higher scores indicating greater anticipated discrimination. In the current sample, the InDI-A measure had a Cronbach’s alpha of 0.87, indicating excellent reliability.

The survey also included questions on sociodemographic characteristics (i.e., race, gender), English proficiency (as defined by speaking English “very well,” “well,” “not well,” or “not at all” by self-report), health care access, the impact of the COVID-19 pandemic, and vaccine hesitancy.

### Statistical analysis

First, we analyzed the baseline characteristics of the sample, the prevalence of a positive screen for anxiety, depression, or alcohol use disorder, and the InDI-A score. We also calculated the proportion of participants that strongly agreed or agreed with each of the nine questions in the InDI-A. Categorical variables were presented as percentages and proportions while continuous data was summarized as mean and standard deviation (SD). Next, three multivariate logistic regression models were constructed with GAD-2, PHQ-2, and AUDIT-C as dependent variables. To isolate the independent association between anticipated discrimination (InDI-A score) and mental health outcomes, each model was adjusted for all other variables. Two participants did not respond to the screening tool for anxiety, 9 for depression, and 54 for hazardous alcohol use screening.

To assess the dosage effect of the overall InDI-A score and each anticipated discrimination item on mental health outcomes, we conducted logistic regression models, adjusted for all other variables. For this analysis, the overall InDI-A was computed and subsequently presented in quartiles, and the individual item questions were categorized based on the 5-point Likert scale. All analyses were performed in R version 4.2.3.

## Results

The sociodemographic characteristics of study participants (*N* = 810) are presented in Table 1. Notably, the study sample was primarily uninsured (66.7%, *N* = 540), with low educational levels (60.7% reported attainment less than high school completion, *N* = 491), and low household incomes (48.9% had household incomes less than $35,000 per year; *N* = 396). More than a third (36.7%, *N* = 297) of participants reported limited English proficiency (“not well” or “not at all”).

**Table 1.** Study Participant Characteristics (*n* = 810)

Characteristic (*n*, %)	Total (*n* = 810)	Men (*n* = 292)	Female (*n* = 518)
Age, years (mean [SD])	39.9 (12.3)	39.3 (12.0)	40.2 (12.4)
Gender			
Male	292 (36.0)	—	—
Female	518 (64.0)	—	—
Race			
White	83 (10.2)	22 (7.5)	61 (11.8)
Black or African American	12 (1.5)	7 (2.4)	5 (1.0)
Native Hawaiian or other Pacific Islander	1 (0.1)	0 (0)	1 (0.2)
Native American or Native Alaskan	2 (0.2)	1 (0.3)	1 (0.2)
Asian	0 (0)	0 (0)	0 (0)
Other	423 (52.2)	169 (57.9)	254 (49.0)
Prefer not to answer	289 (35.6)	93 (31.8)	195 (37.6)
Family income			
≥35,000	98 (12.1)	60 (20.5)	38 (7.3)
<35,000	396 (48.9)	134 (46.9)	262 (50.6)
Preferred not to answer	316 (39.0)	98 (33.6)	218 (42.1)
Education			
High school graduate/GED	319 (39.4)	107 (36.6)	212 (40.9)
Some high school	203 (25.1)	77 (26.4)	126 (24.3)
8th grade or less	288 (35.6)	108 (37.0)	180 (34.7)
English proficiency			
Report speaking English “well” or “very well”	429 (53.0)	143 (49.0)	286 (55.2)
Report speaking English “not well” or “not at all”	297 (36.7)	118 (40.4)	179 (34.6)
Missing	84 (10.4)	31 (10.6)	53 (10.2)
Region of birth			
Mexico	310 (38.3)	133 (45.5)	177 (34.2)
Central America	378 (46.7)	116 (39.7)	262 (50.6)
Caribbean	75 (9.3)	29 (9.9)	46 (8.8)
South America	47 (5.8)	14 (4.8)	33 (6.4)
Health insurance			
Insured	270 (33.3)	99 (33.9)	171 (33.0)
Uninsured	540 (66.7)	193 (66.1)	347 (67.0)
Primary care physician			
Yes	337 (41.6)	96 (32.9)	241 (46.5)
No	473 (58.4)	196 (67.1)	277 (53.5)
Anxiety symptoms (GAD-2)	*N* = 808	*N* = 291	*N* = 517
<3	604 (74.8)	234 (80.4)	370 (71.6)
≥3	204 (25.2)	57 (19.6)	147 (28.4)
Depression symptoms (PHQ-2)	*N* = 801	*N* = 286	*N* = 515
<3	656 (81.9)	238 (83.2)	418 (81.2)
≥3	145 (18.1)	48 (16.8)	97 (18.8)
Hazardous alcohol (AUDIT-C)	*N* = 756	*N* = 270	*N* = 486
No	603 (79.8)	175 (64.8)	428 (88.1)
Yes (≥4 for men; ≥3 for women)	153 (20.2)	95 (35.2)	58 (11.9)

AUDIT-C, Alcohol Use Disorders Identification Test—Concise; GAD, generalized anxiety disorder; GED, General Education Diploma; PHQ, Patient Health Questionnaire; SD, standard deviation.

The mean InDI-A score was 13.2 (SD = 9.1). A high proportion of participants anticipated being the target of multiple types of discrimination (Table 2), with almost half (47.8%, *N* = 387) anticipating work-related discrimination by a supervisor, 42.8% (*N* = 347) potential discrimination when looking for employment, and 40.9% (*N* = 331) when looking for housing. A third of participants (34.1%, *N* = 276) anticipated being treated poorly in health care settings and 40.1% (*N* = 325) were concerned about police harassment. Approximately a third of the respondents thought they would be denied banking services (35.2%, *N* = 285), harassed in public (29.8%, *N* = 241), or become victims of violence (29.0%, *N* = 235) due to their identity.

**Table 2.** Distribution of “Agree or Strongly Agree” Responses in the Intersectional Anticipated Discrimination (InDI-A) Scale

Discrimination type	Question	Total (*n* = 810)	Male (*n* = 292)	Female (*n* = 518)
Health care	Because of who I am, a doctor or nurse, or other health care provider might treat me poorly.	276 (34.1)	89 (30.5)	187 (36.1)
Employment	Because of who I am, I might have trouble finding or keeping a job.	347 (42.8)	121 (41.4)	226 (43.6)
Housing	Because of who I am, I might have trouble getting an apartment or house.	331 (40.9)	124 (42.5)	207 (40.0)
Supervisor	I worry about being treated unfairly by a teacher, supervisor, or employer.	387 (47.8)	136 (46.6)	251 (48.5)
Banking	I may be denied a bank account, loan, or mortgage because of who I am.	285 (35.2)	105 (36.0)	180 (34.7)
Police	I worry about being harassed or stopped by police or security.	325 (40.1)	123 (42.1)	202 (39.0)
Violence	Because of who I am, people might try to attack me physically.	235 (29.0)	91 (31.2)	144 (27.8)
Harassment	I expect to be pointed at, called names, or harassed when in public.	241 (29.8)	96 (32.9)	145 (28.0)
Total InDI-A Score (mean, SD)		13.2 (9.1)	13.1 (9.0)	13.3 (9.2)

InDI-A, intersectional anticipated discrimination index; SD, standard deviation.

Anxiety, stress, and unhealthy alcohol use were also common. Approximately one-quarter (25.2%, *N* = 204/808) of our study population screened positive for anxiety symptoms, 18.1% (*N* = 145/801) for depression symptoms, and 20.2% (*N* = 153/756) for hazardous alcohol use. Out of 747 participants who answered all the mental health related questions, only 4.2% (*N* = 32/747) screened positive for symptoms of all three mental health outcomes, and 43.1% (*N* = 322/747) of participants screened positive for at least one condition.

Findings from the multivariate logistic regression models on factors associated with a positive screen for adverse mental health outcomes are shown in Table 3. A positive screen for generalized anxiety was associated with female gender (adjusted odds ratio [AOR]: 1.8, 95% confidence interval [CI]: 1.2, 2.6), low educational attainment (AOR: 1.6, 95% CI: 1.0, 2.4), and higher anticipated discrimination (AOR: 1.05, 95% CI: 1.03, 1.07). A positive screening for depression was associated with low educational attainment (AOR: 1.9, 95% CI: 1.2, 3.1) and higher anticipated discrimination (AOR: 1.05, 95% CI: 1.03, 1.07). Females were less likely to screen positive for hazardous alcohol use compared with men (AOR: 0.2, 95% CI: 0.2, 0.4), while participants from the Caribbean had a higher risk of hazardous alcohol use (AOR: 2.1, 95% CI: 1.1, 4.1). English proficiency was associated with hazardous alcohol only in univariate analysis, but not after adjustment, and was not associated with anxiety or depression.

**Table 3.** Factors Associated with Symptoms of Anxiety, Depression, or Hazardous Alcohol Use

Variable	PHQ-2 (*n* = 801)		GAD-2 (*n* = 808)		AUDIT-C (*n* = 756)	
	OR (95% CI)	AOR (95% CI)	OR (95% CI)	AOR (95% CI)	OR (95% CI)	AOR (95% CI)
Age	1.01 (0.99, 1.02)	1.00 (0.98, 1.01)	1.00 (0.99, 1.02)	0.99 (0.98, 1.01)	1.00 (0.99, 1.02)	1.00 (0.98, 1.01)
Gender						
Male	1.0	1.0	1.0	1.0	1.0	1.0
Female	1.2 (0.8, 1.7)	1.05 (0.7, 1.6)	1.6 (1.2, 2.3)^**^	1.8 (1.2, 2.6)^**^	0.2 (0.2, 0.4)^***^	0.2 (0.2, 0.4)^***^
Family Income						
≥ 35,000	1.0	1.0	1.0	1.0	1.0	1.0
< 35,000	1.5 (0.8, 2.9)	1.5 (0.7, 3.0)	1.0 (0.6, 1.6)	0.9 (0.5, 1.5)	0.6 (0.4, 1.0)	1.0 (0.6, 1.7)
No response	1.8 (0.9, 3.5)	2.4 (1.2, 4.9)^*^	0.7 (0.4, 1.2)	0.9 (0.5, 1.6)	0.3 (0.2, 0.5)^***^	0.5 (0.3, 0.8)^*^
Education						
High school graduate/GED	1.0	1.0	1.0	1.0	1.0	1.0
Some high school	1.3 (0.8, 2.1)	1.2 (0.7, 1.9)	1.5 (1.0, 2.2)	1.4 (0.9, 2.1)	1.5 (1.0, 2.3)	1.3 (0.8, 2.1)
8^th^ grade or less	1.7 (1.1, 2.6)^*^	1.9 (1.2, 3.1)^**^	1.2 (0.8, 1.7)	1.6 (1.0, 2.4)^*^	1.0 (0.7, 1.6)	1.2 (0.8, 2.0)
English proficiency						
Good	1.0	1.0	1.0	1.0	1.0	1.0
Limited	0.9 (0.6, 1.3)	1.0 (0.6, 1.5)	1.2 (0.9, 1.7)	1.1 (0.8, 1.7)	1.6 (1.1, 2.4)^*^	1.4 (0.9, 2.2)
No response	1.0 (0.6, 1.9)	1.0 (0.5, 2.0)	1.4 (0.9, 2.4)	1.2 (0.7, 2.2)	2.7 (1.6, 4.6)^***^	2.0 (1.1, 3.7)^*^
Region of birth						
Mexico	1.0	1.0	1.0	1.0	1.0	1.0
Central America	1.2 (0.8, 1.9)	1.2 (0.8, 1.8)	1.2 (0.9, 1.8)	1.2 (0.8, 1.7)	0.6 (0.4, 1.0)	0.7 (0.5, 1.1)
Caribbean	2.3 (1.3, 4.1)^**^	2.2 (1.1, 4.3)^*^	4.0 (2.4, 6.8)	3.0 (1.6, 5.5)^***^	2.7 (1.6, 4.7)	2.1 (1.1, 4.1)^*^
South America	1.0 (0.4, 2.3)	1.01 (0.4, 2.5)	1.3 (0.7, 2.7)	1.2 (0.6, 2.7)	0.6 (0.2, 1.4)	0.5 (0.2, 1.4)
Health insurance						
Insured	1.0	1.0	1.0	1.0	1.0	1.0
Uninsured	0.7 (0.5, 1.0)^*^	0.7 (0.4, 1.2)	0.5 (0.4, 0.8)^***^	0.7 (0.4, 1.0)	0.6 (0.4, 0.9)^*^	1.1 (0.6, 1.9)
PCP						
Yes	1.0	1.0	1.0	1.0	1.0	1.0
No	0.7 (0.5, 0.9)^*^	0.7 (0.4, 1.2)	0.6 (0.4, 0.8)^**^	0.9 (0.6, 1.4)	0.8 (0.5, 1.1)	0.7 (0.4, 1.2)
InDI-A score	1.05 (1.0, 1.07)^***^	1.05 (1.03, 1.07)^***^	1.05 (1.03, 1.07)^***^	1.05 (1.03, 1.07)^***^	1.00 (0.99, 1.03)	1.00 (0.98, 1.02)

^*^ *p* < 0.05.

^**^ *p* < 0.01.

^***^ *p* < 0.001.

AORs, adjusted for age, gender, income, education, English proficiency, discrimination score, region of birth, Insurance, and PCP.

AOR, adjusted odds ratio; AUDIT-C, Alcohol Use Disorders Identification Test—Concise; CI, confidence interval; GAD, generalized anxiety disorder; GED, general education diploma; InDI-A, Intersectional Anticipated Discrimination Index; OR, odds ratio; PCP, primary care provider; PHQ, Patient Health Questionnaire.

**FIG. 1.**
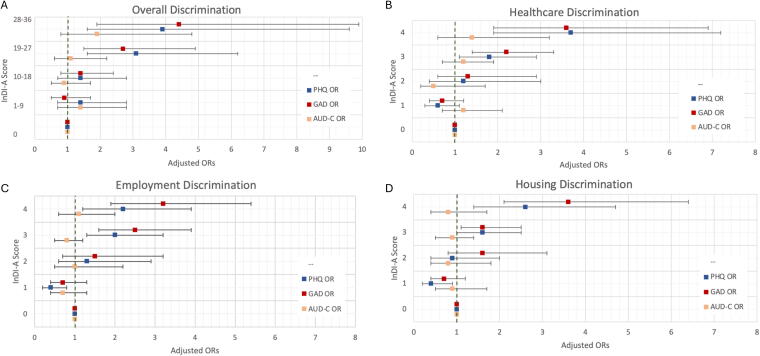
Association of Intersectional Anticipated Discrimination Scale (InDI-A) scores and positive mental health screen. Adjusted odds ratios (AORs) for the association between anticipated discrimination (InDI-A) positive screening for Generalized Anxiety Disorder 2-item (GAD). Patient Health Questionnaire-2 (PHQ). Alcohol Use Disorder Identification Test—Concise (AUDIT-C). **(A)** Overall discrimination scale scores (by quartile). Responses to questions about health care **(B)**, employment **(C)**, and housing **(D)** using the Likert scale (0 = Strongly disagree, 1 = Disagree, 2 = Neither agree nor disagree, 3 = Agree, 4 = Strongly agree).

A dose-response relationship was observed between the InDI-A score and the likelihood of screening positive for anxiety and depression (Fig. 1). Participants with the highest quartile overall InDI-A score had approximately four times higher likelihood of anxiety (AOR: 4.4, 95% CI: 1.9, 9.9) or depression symptoms (AOR: 3.9, 95% CI: 1.6, 9.6) compared with those with the lowest quartile. When examining responses by domains, we found a dose–response relationship between anxiety or depression and discrimination in health care, employment, and housing (Fig. 1). Participants who strongly agreed with questions related to anticipatory discrimination in health care were more than three times as likely to screen positive for anxiety (AOR: 3.6, 95% CI: 1.9, 6.9) and depression (AOR: 3.7, 95% CI: 1.9, 7.2) than those who strongly disagreed. Those who strongly agreed with questions related to anticipatory discrimination in employment were more than three times as likely to screen positive for anxiety (AOR: 3.2, 95% CI: 1.9, 5.4) and more than twice as likely to screen positive for depression (AOR: 2.2, 95% CI: 1.2, 3.9) than those who strongly disagreed. Likewise, participants who strongly agreed with questions related to anticipatory discrimination in housing were more than three times as likely to screen positive for anxiety (AOR: 3.6, 95% CI: 2.1, 6.4) and more than twice as likely to screen positive for depression (AOR: 2.6, 95% CI: 1.4, 4.7). The association between symptoms of anxiety and depression among participants who “agreed” with statements of anticipatory discrimination in these domains was lower than among those who “strongly agreed” but higher than among those who disagreed with these statements (Fig. 1).

Anticipatory police discrimination, public harassment, and banking discrimination were associated with symptoms of anxiety (AOR_police_: 2.7, 95% CI: 1.6, 4.6; AOR_harassment_: 2.7, 95% CI: 1.5, 4.9; AOR_banking_: 2.2, 95% CI: 1.2, 4.0) or depression (AOR_police_: 2.5, 95% CI: 1.4, 4.5; AOR_harassment;_ 3.0, 95% CI: 1.6; AOR_banking_: 2.1, 95% CI: 1.1, 3.9) only among participants who strongly agreed with these statements. Participants who strongly agreed with statements related to relationship discrimination had a higher likelihood of screening positive for depression (AOR: 2.9, 95% CI: 1.1, 7.4), and those strongly agreeing with statements about physical assault had a higher likelihood of anxiety symptoms (AOR: 2.0, 95% CI: 1.0, 3.7). There was no association between hazardous alcohol consumption and overall InDI-A scores or InDI-A responses in each domain.

## Discussion

In this sample of Latino immigrants, we found that intersectional anticipated discrimination was associated with symptoms of anxiety and depression, but not hazardous alcohol use, in a dose-respondent manner. Given the heightened vigilance and stress that can result from anticipatory discrimination,^15^ the association between intersectional anticipated discrimination and anxiety or depression is perhaps not surprising, but the strength of the relationship is notable. We found that the likelihood of screening positive for depression and anxiety was two to four times higher among participants who strongly agreed with statements related to health care, housing, employment, public harassment, and police discrimination. Although evidence supporting the association between discrimination and adverse mental health outcomes is growing, there is limited understanding of how discrimination affects Latinos with intersecting identities, particularly those who are immigrants. To our knowledge, this is the first quantitative study examining the association between anticipated intersectional discrimination and mental health outcomes in Latino immigrants.

Latinos in the United States are a heterogeneous group with diverse experiences depending on country of origin, race, socioeconomic status, language proficiency, and migration status, among others.^22^ Our study is notable for recruiting a population of immigrant Latinos primarily from Mexico and Central America with low educational attainment and socioeconomic status. Based on community feedback and to promote trust, we did not ask about migration status, but the high proportion of low-income participants without health insurance in Maryland, a Medicaid expansion state, suggests that our sample had high representation of undocumented immigrants (who are ineligible for subsidized health care coverage).

Our study population reported high levels of anticipated intersectional discrimination across all domains, likely reflecting racism, systematic exclusion from economic opportunities, ethnic profiling, xenophobic attitudes, language discrimination, and anti-immigrant policies, among others. Latino immigrants, particularly those who are undocumented, are especially vulnerable to discrimination and violence in the workplace, public spaces, health care, and other domains.^2,23,24^ Concerns about deportation often dissuade victims of discrimination and violence from reporting incidents to authorities, allowing perpetrators to act with impunity.^2^ Furthermore, anti-immigrant policies and the prevailing climate can significantly impact the health and well-being of Latinos, irrespective of citizenship status. Apart from creating a “chilling effect” on health care and service utilization,^25^ these policies can induce stress for both Latino immigrants and those born in the United States, leading to heightened perceptions of discrimination.^8,26^ Overall, the expectation of being discriminated against, coupled with the fear of reporting incidents, has a profound impact on the well-being and mental health of Latinos.

It is worth noting that despite the numerous structural challenges faced by Latino immigrants with language barriers,^1,6,24^ no association was observed between mental health outcomes and limited English proficiency. This is consistent with other studies showing that Latino immigrants who have resided in the United States for longer periods and are more acculturated exhibit higher levels of anxiety and depression.^27^ The reasons behind these findings remain unclear, but certain protective factors that grant resilience to Latino immigrants could play a role. These might include strong social connections, the specific characteristics of individuals who choose to migrate, and differing concepts of mental health among Latino immigrants of varying socioeconomic and educational backgrounds.^28^ In fact, among Latino immigrants, citizenship, and discrimination appear to be more strongly associated with psychological distress than English language proficiency.^1,29^

Our study had a few unexpected findings. We found no clear correlation between health care access and mental health. This could be attributed to various factors. Barriers such as language and health insurance can impede access to mental health services. However, even after accounting for these factors, Latinos with limited English proficiency are less likely to utilize mental health services, suggesting that cultural differences or stigma may play a role and potentially lead to underreporting of symptoms.^30^ In addition, there was no association between anticipated discrimination—overall or by domain—and hazardous alcohol. Prior studies have reported such associations among gender and sexual minority Latino men,^31,32^ as well as in heterosexual Latino men, but not women.^33^ In our study, almost two-thirds of participants were women, which may have influenced our results and may reflect different intersecting disadvantaged identities among study populations.

Our findings should be interpreted in light of several limitations. Causal inferences cannot be made from this cross-sectional design study and the relationship between discrimination and mental health conditions may be bidirectional. We were unable to assess race due to a high number of missing or other responses, which is a well-documented challenge when applying U.S.-centric racial categories to Latinos.^34,35^ In addition, the InDI-A measure does not ask about specific identities or how they intersect; therefore, we are unable to directly examine how the convergence of multiple identities may compound effects on mental health and substance misuse outcomes.

In conclusion, both depression and anxiety symptoms are more frequently found among Latino immigrants who experience heightened vigilance to potential experiences of discrimination. These findings are concerning given the increasingly hostile climate for Latino immigrants and the limited availability of mental health services for this population.

## Health Equity Implications

Our study underscores several significant implications for health equity. First, an intersectional framework is essential for understanding and designing effective approaches to mental health promotion and intervention among the immigrant Latino community, as it considers their varied identities encompassing race, ethnicity, immigration status, gender, and socioeconomic background. In health research, Latinos are often treated as a homogenous group despite the wide range of diversity within this population. Effective approaches to mental health promotion and intervention must be responsive to the diverse backgrounds and experiences among Latinos. Health care workers should be educated about this diversity and aware of the impacts of discrimination and anticipated discrimination on the mental health of Latinos, regardless of immigration status or English language proficiency. Latinos are more likely to prefer addressing mental health concerns in primary care,^36^ and thus, this awareness is important for all health care providers, not only those in mental health care. Ultimately, prioritizing culturally competent care, enhancing community support, and implementing targeted policy interventions are imperative steps toward promoting mental health equity among this population.

## Abbreviations Used

AOR: Adjusted Odds Ratio
AUDIT-C: Alcohol Used Disorder Identification Test Condensed
CI: Confidence Interval
COVID-19: Coronavirus Disease 2019
DACA: Deferred Action for Childhood Arrivals
GAD-2: Generalized Anxiety Disorder 2 Item
InDI-A: Intersectional Anticipated Discrimination Scale
N: number
PHQ-2: Patient Health Questionnaire 2 Item
RADx-UP: Rapid Acceleration of Diagnostics-Underserved Populations
SD: Standard Deviation
US: United States
