# Supplementary material for: Association of Intersectional Anticipated Discrimination with Mental Health Among Immigrant Latinos
Source: Health Equity. 2024 Nov 26;8(1):770–9. doi: 10.1089/heq.2024.0072 (PMC11844664; doi:10.1089/heq.2024.0072)
Supplement: Supplementary Table S1 [file heq.2024.0072_supp_tables1.docx]

**Supplemental Table. Association of Intersectional Anticipated Discrimination Scale (InDI-A) scores and positive mental health screen.** Adjusted odds ratios (ORs) for the association between anticipated discrimination (InDI-A) positive screening for Generalized Anxiety Disorder 2-item (GAD). Patient Health Questionnaire-2 (PHQ). Alcohol Use Disorder Identification Test - Concise (AUDIT-C).

|  | PHQ-2 | | GAD-2 | | AUDIT-C | |
| --- | --- | --- | --- | --- | --- | --- |
| InDI-A Score | N | AOR [95% CI] | N | AOR [95% CI] | N | AOR [95% CI] |
| 0 | 113 | 1.0 | 115 | 1.0 | 109 | 1.0 |
| 1-9 | 204 | 1.4 [0.7, 2.8] | 207 | 0.9 [0.5, 1.7] | 190 | 1.4 [0.7, 2.8] |
| 10-18 | 248 | 1.4 [0.7, 2.8] | 248 | 1.4 [0.8, 2.4] | 232 | 0.9 [0.5, 1.7] |
| 19-27 | 194 | 3.1 [1.6, 6.2] ** | 196 | 2.7 [1.5, 4.9] *** | 186 | 1.1 [0.6, 2.2] |
| 28-36 | 42 | 3.9 [1.6, 9.6] ** | 42 | 4.4 [1.9, 9.9] *** | 39 | 1.9 [0.8, 4.8] |
| Healthcare Question |  |  |  |  |  |  |
| Strongly Disagree | 332 | 1.0 | 339 | 1.0 | 321 | 1.0 |
| Disagree | 153 | 0.6 [0.3, 1.1] | 154 | 0.7 [0.4, 1.2] | 140 | 1.2 [0.7, 2.1] |
| Neither Agree nor Disagree | 40 | 1.2 [0.4, 3.0] | 40 | 1.3 [0.6, 2.9] | 36 | 0.5 [0.2, 1.7] |
| Agree | 224 | 1.8 [1.1, 2.9] * | 224 | 2.2 [1.4, 3.3] *** | 214 | 1.2 [0.7, 1.9] |
| Strongly Agree | 52 | 3.7 [1.9, 7.2] *** | 51 | 3.6 [1.9, 6.9] *** | 45 | 1.4 [0.6, 3.2] |
| Employment Question |  |  |  |  |  |  |
| Strongly Disagree | 253 | 1.0 | 257 | 1.0 | 243 | 1.0 |
| Disagree | 152 | 0.4 [0.2, 0.8] ** | 153 | 0.7 [0.4, 1.3] | 139 | 0.7 [0.4, 1.3] |
| Neither Agree nor Disagree | 53 | 1.3 [0.6, 2.9] | 53 | 1.5 [0.7, 3.2] | 49 | 1.0 [0.5, 2.2] |
| Agree | 240 | 2.0 [1.3, 3.2] ** | 240 | 2.5 [1.6, 3.9] *** | 224 | 0.8 [0.5, 1.2] |
| Strongly Agree | 103 | 2.2 [1.2, 3.9] ** | 105 | 3.2 [1.9, 5.4] *** | 101 | 1.1 [0.6, 2.0] |
| Housing Question |  |  |  |  |  |  |
| Strongly Disagree | 274 | 1.0 | 278 | 1.0 | 261 | 1.0 |
| Disagree | 143 | 0.4 [0.2, 0.9] * | 142 | 0.7 [0.4, 1.2] | 131 | 0.9 [0.5, 1.7] |
| Neither Agree nor Disagree | 58 | 0.9 [0.4, 2.0] | 58 | 1.6 [0.8, 3.1] | 55 | 0.8 [0.4, 1.8] |
| Agree | 247 | 1.6 [1.0, 2.5] | 249 | 1.6 [1.1, 2.5] * | 232 | 0.9 [0.5, 1.4] |
| Strongly Agree | 79 | 2.6 [1.4, 4.7] ** | 81 | 3.6 [2.1, 6.4] *** | 77 | 0.8 [0.4, 1.7] |
| Supervisor Question |  |  |  |  |  |  |
| Strongly Disagree | 242 | 1.0 | 246 | 1.0 | 232 | 1.0 |
| Disagree | 119 | 0.6 [0.3, 1.3] | 118 | 1.0 [0.5, 1.8] | 109 | 0.6 [0.3, 1.2] |
| Neither Agree nor Disagree | 58 | 0.5 [0.2, 1.6] | 57 | 1.1 [0.5, 2.4] | 53 | 0.4 [0.2, 1.1] |
| Agree | 271 | 2.0 [1.2, 3.2] ** | 275 | 2.4 [1.5, 3.7] *** | 257 | 1.0 [0.6, 1.6] |
| Strongly Agree | 111 | 2.8 [1.6, 4.9] *** | 112 | 3.7 [2.2, 6.3] *** | 105 | 1.1 [0.6, 2.1] |
| Banking Question |  |  |  |  |  |  |
| Strongly Disagree | 303 | 1.0 | 309 | 1.0 | 293 | 1.0 |
| Disagree | 145 | 0.7 [0.4, 1.3] | 145 | 0.6 [0.4, 1.1] | 129 | 1.0 [0.5, 1.8] |
| Neither Agree nor Disagree | 71 | 0.5 [0.2, 1.2] | 70 | 1.0 [0.5, 1.8] | 66 | 0.9 [0.4, 1.8] |
| Agree | 216 | 1.3 [0.8, 2.1] | 217 | 1.5 [1.0, 2.2] | 207 | 1.3 [0.8, 2.0] |
| Strongly Agree | 66 | 2.1 [1.1, 3.9] * | 67 | 2.2 [1.2, 4.0] ** | 61 | 1.0 [0.5, 2.1] |
| Police Question |  |  |  |  |  |  |
| Strongly Disagree | 284 | 1.0 | 290 | 1.0 | 280 | 1.0 |
| Disagree | 130 | 0.7 [0.4, 1.3] | 129 | 0.5 [0.3, 0.9] * | 120 | 0.9 [0.5, 1.7] |
| Neither Agree nor Disagree | 65 | 0.3 [0.1, 1.0] * | 65 | 0.7 [0.3, 1.3] | 56 | 0.9 [0.4, 1.9] |
| Agree | 230 | 1.4 [0.8, 2.1] | 231 | 1.2 [0.8, 1.8] | 213 | 1.0 [0.6, 1.6] |
| Strongly Agree | 92 | 2.5 [1.4, 4.5] ** | 93 | 2.7 [1.6, 4.6] *** | 87 | 1.1 [0.6, 2.1] |
| Harassment Question |  |  |  |  |  |  |
| Strongly Disagree | 324 | 1.0 | 328 | 1.0 | 311 | 1.0 |
| Disagree | 168 | 0.7 [0.4, 1.2] | 167 | 0.9 [0.5, 1.4] | 155 | 0.9 [0.5, 1.5] |
| Neither Agree nor Disagree | 73 | 0.9 [0.4, 1.9] | 73 | 1.3 [0.7, 2.4] | 67 | 0.5 [0.2, 1.1] |
| Agree | 174 | 1.6 [1.0, 2.5] | 176 | 1.2 [0.8, 1.8] | 162 | 1.2 [0.7, 1.9] |
| Strongly Agree | 62 | 3.0 [1.6, 5.6] *** | 64 | 2.7 [1.5, 4.9] ** | 61 | 0.8 [0.4, 1.7] |
| Assault Question |  |  |  |  |  |  |
| Strongly Disagree | 343 | 1.0 | 348 | 1.0 | 327 | 1.0 |
| Disagree | 154 | 0.6 [0.3, 1.0] | 154 | 0.8 [0.5, 1.3] | 142 | 0.9 [0.6, 1.4] |
| Neither Agree nor Disagree | 72 | 0.8 [0.4, 1.7] | 71 | 1.2 [0.6, 2.1] | 66 | 0.7 [0.3, 1.4] |
| Agree | 178 | 1.3 [0.8, 2.0] | 181 | 1.0 [0.7, 1.6] | 171 | 1.0 [0.6, 1.7] |
| Strongly Agree | 54 | 1.5 [0.7, 3.1] | 54 | 2.0 [1.0, 3.7] * | 50 | 1.4 [0.7, 2.9] |
| Relationship Question |  |  |  |  |  |  |
| Strongly Disagree | 469 | 1.0 | 475 | 1.0 | 440 | 1.0 |
| Disagree | 163 | 0.6 [0.4, 1.0] | 163 | 0.8 [0.5, 1.2] | 153 | 0.7 [0.4, 1.2] |
| Neither Agree nor Disagree | 69 | 0.5 [0.2, 1.2] | 68 | 1.0 [0.6, 1.9] | 63 | 0.6 [0.3, 1.4] |
| Agree | 78 | 1.4 [0.8, 2.4] | 80 | 1.7 [1.0, 2.9] | 76 | 1.1 [0.6, 2.1] |
| Strongly Agree | 22 | 2.9 [1.1, 7.4] * | 22 | 2.5 [1.0, 6.4] | 20 | 1.5 [0.5, 4.3] |

*p<.05, ** p<.01, ***p<.001

OR= odds ratio, AOR= adjusted odds ratio, CI= Confidence Interval, GAD= Generalized Anxiety Disorder, PHQ= Patient Health Questionnaire, AUDIT-C= Alcohol Use Disorders Identification Test- Concise, InDI-A= Intersectional Anticipated Discrimination Index

AORs adjusted for age, gender, income, education, English proficiency, discrimination Score, Region of Birth, Insurance and PCP
